# Multiomics Identifies Potential Biomarkers in Ankylosing Spondylitis Bone Formation

**DOI:** 10.1155/humu/8771129

**Published:** 2025-08-08

**Authors:** Lu Yang, Chunping Bo, Meiqi Chen, Bozhen Chen, Rui Zeng, Yingyan Zhou, Haifang Du, Xiaohong He

**Affiliations:** ^1^Second Clinical Medical College, Guangzhou University of Chinese Medicine, Guangzhou, Guangdong Province, China; ^2^Department of Rheumatology, Guangdong Provincial Hospital of Chinese Medicine, Guangzhou, Guangdong Province, China; ^3^Guangdong Provincial Key Laboratory of Clinical Research on Traditional Chinese Medicine Syndrome, Guangdong Provincial Hospital of Chinese Medicine, Guangzhou, Guangdong Province, China

**Keywords:** AS, machine learning, MR, transcriptomic analysis

## Abstract

**Objective:** Ankylosing spondylitis (AS) is a long-term inflammatory condition characterized by intricate pathogenesis and significant genetic predisposition. Current treatment methods cannot completely halt the progression of the disease. The purpose of this research is to discover possible therapeutic targets for AS by integrating Mendelian Randomization (MR), transcriptomics analysis, and machine learning, providing new options for the clinical treatment of AS.

**Methods:** In this study, we initially pinpointed differentially expressed genes (DEGs) linked to AS from the GEO database and acquired cis-eQTL data for these genes from the eQTLGen Consortium. Using MR and summary data–based Mendelian randomization (SMR) analyses, we screened for DEGs with causal relationships to AS. Subsequently, we analyzed the correlation between these causal genes and immune cell expression, constructed a risk prediction model, and identified key feature genes for AS. Next, we conducted phenome-wide association studies (PheWASs) on the identified AS feature genes to predict their potential adverse effects as therapeutic targets. We obtained AS-related therapeutic drugs from the DrugBank database and performed molecular docking analysis with AS feature genes. We used the CAIA collagen-induced AS mouse model; we measured joint swelling and employed microCT, H&E, and Safranin O-Fast Green staining to assess pathological changes in bone tissue. Additionally, we employed Western blot and RT-qPCR to analyze the expression levels of genes associated with bone mineralization and AS feature genes in joint tissues.

**Results:** A total of 1607 DEGs were obtained from the GEO database. After MR analysis and correction, 33 positive DEGs that have a causal relationship with AS were determined. Through the correlation analysis between these genes and the expressions of immune cells, it was found that 28 genes had significant regulatory relationships with 19 kinds of immune cells, with 55 pairs of negative regulatory relationships and 49 pairs of positive regulatory relationships, respectively. Four machine learning model algorithms determined the Top 5 genes (RIOK1, FUCA2, COL9A2, USP16, and TTC16) with the highest importance scores and constructed a nomogram to evaluate the risk probability. The results of the PheWAS showed that the five characteristic genes of AS had harmful or beneficial effects on numerous disease phenotypes of multiple types of diseases. Molecular docking indicated that 14 known AS treatment drugs had potential interactions with related genes. Using RT-qPCR, we evaluated the expression levels of five key genes in the joint tissue of the CAIA collagen-induced AS mouse model. Compared to the normal control group, we found that the levels of *FUCA2* and *USP16* were significantly elevated, while the levels of *TTC16* were significantly reduced. In contrast, the expression of *COL9A2* and *RIOK1* mRNA showed no significant difference.

**Conclusion:** Our research findings demonstrate that FUCA2, USP16, and TTC16 may serve as biomarkers for AS.

## 1. Introduction

Ankylosing spondylitis (AS) is a chronic joint inflammation disorder with a strong genetic basis, predominantly affecting the spine, sacroiliac joints, and surrounding soft tissues [1]. During the progression of AS, inflammatory processes can induce fibrosis and calcification, ultimately leading to a reduction in spinal flexibility and eventual fusion. The primary clinical manifestations of AS include back pain and spinal stiffness [2]. The global prevalence of AS is estimated to range from 0.07% to 0.31%, with a higher incidence observed in young males [3]. Traditional therapeutic strategies for AS predominantly involve the administration of nonsteroidal anti-inflammatory drugs (NSAIDs), tumor necrosis factor (TNF) inhibitors, and interleukin-17 inhibitors, which have demonstrated efficacy in controlling inflammation and mitigating symptoms. Nonetheless, these treatments neither arrest disease progression nor address the underlying pathophysiological mechanisms of AS, and they are frequently associated with significant adverse effects [4]. Therefore, there is an imperative need to explore more efficacious therapeutic approaches to enhance the quality of life for individuals affected by AS.

Genes are integral to a multitude of biological processes and serve as pivotal targets for pharmacological intervention. Recently, methods such as Mendelian randomization (MR) and machine learning, which are used as approaches for identifying new therapeutic targets, have received considerable attention [5]. This technique employs single nucleotide polymorphisms (SNPs) as instrumental variables to investigate causal relationships between exposures and diseases, effectively simulating randomized controlled trials and thereby providing robust support for the identification of novel therapeutic targets [6, 7]. Additionally, applying phenome-wide association studies (PheWASs) can help predict adverse effects associated with these targets [8].

In this study, we first identified differentially expressed genes (DEGs) related to AS from the Gene Expression Omnibus (GEO) database and obtained *cis*-expression quantitative trait locus (*cis*-eQTL) data for these genes from the eQTLGen Consortium. Using MR and summary Mendelian randomization (SMR) analyses, we filtered out DEGs with causal relationships to AS. Subsequently, we performed correlation analyses between these causally related genes and immune cell expression, constructed a risk prediction model, and identified key AS signature genes. We then conducted PheWAS to predict potential adverse effects when these signature genes are used as therapeutic targets. We retrieved AS-related therapeutic drugs from the DrugBank database and conducted molecular docking analyses with the AS signature genes. Further, we established an AS mouse model and evaluated pathologic ossification through joint swelling assessment, microCT, H&E staining, and Safranin O-Fast Green staining. Western blot was used to detect the expression of pathologic ossification related genes, and PCR was performed to validate the expression of characteristic genes in joint tissues. This study employs an innovative methodology that integrates transcriptomic analysis with genomic MR analysis to explore causal relationships between genes and AS. The objective is to identify potential therapeutic targets for AS and to offer new avenues for the development of AS treatments.

## 2. Materials and Methods

### 2.1. Study Design and Ethics

**Figure 1:**
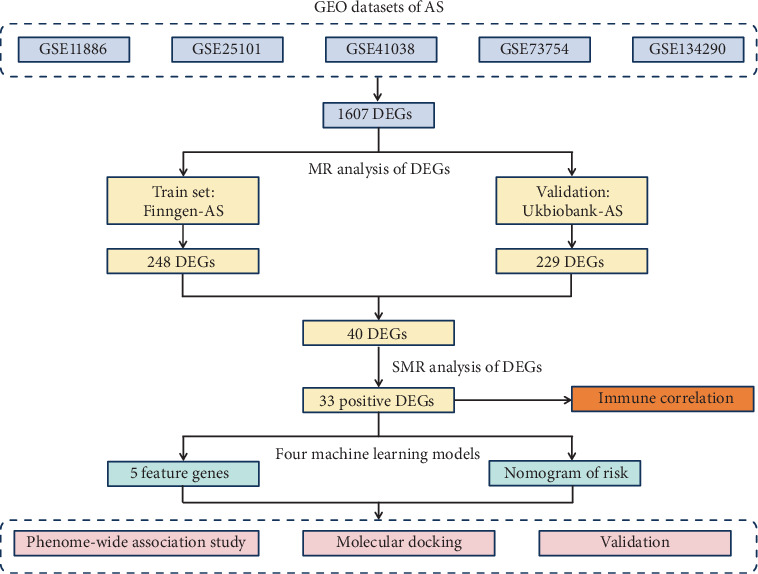
Flowchart of this study design.

The overall study design is illustrated in Figure 1. In this study, we utilized publicly available GEO and genome-wide association study (GWAS) summary data, with all participants providing informed consent and corresponding ethical approvals from the relevant review boards. Animal experiments were approved by the Ethics Committee of Guangdong Provincial Hospital of Traditional Chinese Medicine (Approval No. 2022025).

### 2.2. Data Sources

The human transcriptome data used in this study were obtained from the GEO database (https://www.ncbi.nlm.nih.gov/geo/). On March 25, 2024, we searched the keyword “Ankylosing spondylitis,” specifying the organism as “Homo sapiens” and the data type as “expression profiling by array, real-time polymerase chain reaction, and high-throughput sequencing.” The sample content was RNA expression data, with no intervention involved.

The cis-eQTL data for AS-related DEGs in this study were obtained from the eQTLGen Consortium (https://eqtlgen.org/cis-eqtls.html). The genetic instrumental variables we selected met the following criteria: a *p*-value less than 5 × 10^−8^, minor allele frequency (MAF) greater than 1%, an *F*-statistic value exceeding 10, and the cis-eQTLs located within 1 Mb upstream or downstream of the gene.

The AS GWAS summary data used in this study were obtained from the R10 release of the FinnGen study (https://www.finngen.fi/en/access_results) and the UK Biobank (https://www.ukbiobank.ac.uk/). The FinnGen AS dataset includes 3162 AS patients and 294,770 controls, with cases defined using International Classification of Diseases (ICD) codes, including ICD10-M45, ICD9-7200, and ICD8-7124. The UK Biobank AS dataset contains 1296 AS patients and 461,637 controls, with cases primarily defined by self-reported diagnoses.

The phenotypic data used in this study were obtained from the UK Biobank summary statistics in the IEU (Integrative Epidemiology Unit) database, including a total of 3948 phenotypes involving various disease phenotypes, biochemical markers, and health conditions.

The drug information related to AS in this study was obtained from the DrugBank database (https://go.drugbank.com/). We searched for “Ankylosing spondylitis” in the disease search section, excluding all veterinary drugs, withdrawn drugs, and drugs without available three-dimensional structures. Additionally, we obtained three-dimensional structures of drugs and targets from the Zinc (https://zinc.docking.org/) and PDB (https://www.rcsb.org/) databases.

### 2.3. Identification of AS DEGs

The included AS human RNA expression datasets were batch-combined using the “sva” package in R, and gene symbol annotation was performed using Perl scripts. We then calculated the mean RNA expression levels for both the control and AS groups and performed differential expression analysis. DEGs were selected using *p* value < 0.05 and |logFC| > 1.5. For the genes, statistical significance was assessed with a two-tailed *t*-test.

### 2.4. MR Analysis of AS DEGs

In this study, the "org.Hs.eg.db" package was used to convert DEGs' symbol IDs to Ensembl IDs, and cis-eQTL data for the DEGs were matched from the eQTLGen Consortium as genetic instrumental variables for exposure. The initial number of SNPs obtained from the eQTLGen Consortium was 19,942, filtered using criteria of *p* value < 5 × 10^−8^, MAF > 1%, *F*‐statistic > 10, and proximity within 1 Mb of the gene. Using the “TwoSampleMR” package, we performed MR analysis between these instrumental variables and AS GWAS datasets provided by FinnGen and the UK Biobank. The GWAS data from FinnGen served as the training set, while the GWAS data from the UK Biobank served as the validation set. To ensure the robustness of the results, we considered the intersection of positive results from both datasets as DEGs with causal relationships to AS. MR analysis needs to meet the following conditions: the SNP-exposure association must be strong, independent of confounders, and only influence the outcome through exposure. The latter two assumptions cannot be empirically verified.

In the MR analysis, the random effects inverse-variance weighted (IVW) method was employed. In instances where the genetic instrumental variable for exposure comprised only a single SNP, the Wald ratio method was utilized. SNP effect alleles were harmonized using the “harmonise_data' function in the TwoSampleMR package. Cochran's *Q* statistic was applied to assess heterogeneity within the results, with a threshold of *p* < 0.05 denoting significant heterogeneity. To evaluate horizontal pleiotropy, the MR Egger method was implemented, considering a *p* value of < 0.05 as indicative of significant horizontal pleiotropy. Results demonstrating significant heterogeneity or horizontal pleiotropy were subsequently excluded from further analysis.

The DEGs that were positive in the MR analysis intersection were then subjected to SMR analysis with the training set to further validate the causal relationship between these DEGs and AS. The HEIDI (heterogeneity in dependent instruments) test was used to detect linkage disequilibrium in the results, and when the *p* value of the HEIDI test was ≤ 0.01, linkage disequilibrium was considered significant, and the corresponding results were excluded. Finally, we identified a set of DEGs with a clear causal relationship with AS, defined as positive DEGs for AS. In addition, we analyzed the expression differences of these 33 Positive DEGs between the AS group and the control group to determine the expression levels of these genes in AS patients.

### 2.5. Correlation Analysis Between Positive DEGs and Immune Cell Expression

We visualized the expression differences of AS Positive DEGs between the control and AS groups to evaluate their regulatory relationships with AS. Subsequently, we used the CIBERSORT tool to estimate the relative proportions of immune cells, summing to one. The expression levels of positive DEGs were then correlated with the relative abundance of immune cells using the Pearson correlation coefficient to obtain correlation coefficients, with the results visualized in a heatmap.

### 2.6. Identification of AS Signature Genes and Construction of Machine Learning Models

We constructed four predictive models using the expression data of positive DEGs: a random forest (RF) model, a support vector machine (SVM) model, a generalized linear model (GLM), and an extreme gradient boosting (XGBoost) model. These models were chosen for their complementary strengths: RF for handling high-dimensional data, SVM for robustness to outliers, GLM for interpretability, and XGBoost for predictive accuracy. RF ranked genes by mean decrease in accuracy (MDA) from permutation importance; SVM used radial basis function (RBF) kernel coefficients; GLM applied LASSO regularization with *λ* selected via 10-fold cross-validated AUC optimization; XGBoost employed feature gain metrics. All models underwent 10-fold stratified cross-validation with AUC-ROC as the primary performance metric. After calculating the predictive functions, we selected the most suitable predictive model and identified signature genes from the DEGs through a comprehensive analysis involving residual boxplots and receiver operating characteristic (ROC) curves. Subsequently, a nomogram was created based on the expression of signature genes in both the control and AS groups. To evaluate the accuracy and generalization ability of the nomogram, we generated decision curves and calibration curves.

### 2.7. PheWAS of AS Signature Genes

To evaluate the potential side effects of AS signature genes as potential drug targets, we conducted PheWAS on these signature genes across 3948 phenotypes in the UK Biobank database. This analysis is aimed at exploring the potential beneficial or harmful effects of these signature genes on other diseases. The MR analysis followed the same criteria as previously described.

### 2.8. Molecular Docking of AS Signature Genes

We obtained AS related therapeutic drugs from DrugBank and performed molecular docking using Autodock Vina v1.2.0. A docking score of less than −5.0 kcal/mmol was considered indicative of a stable docking structure, which allowed us to preliminarily assess the interactions between AS-related therapeutic drugs and signature genes. The Top 4 docking combinations were selected and visualized using PyMOL v2.5.2.

### 2.9. Experimental Animals and Model Establishment

SPF-grade BALB/c male mice, 8 weeks old (body weight 20 ± 2 g), were purchased from Guangzhou Bioenergy Co., Ltd. (Certificate No.44829700012174). The mice were acclimatized for 1 week under standard laboratory conditions. They were randomly divided into a normal group and a model group, with 6 mice per group. The model group was induced to develop an AS model. The specific modeling procedure was as follows: On the first day of the experiment, mice were injected via the tail vein with 2 mg/kg of Arthrogen-CIA 5-Clone Cocktail Kit (Chondrex, 53100). On Day 7, they received an injection of 50 *μ*g/mouse of Biotinylated Lipopolysaccharide (Chondrex, 9028). The control group received an equivalent volume of saline via tail vein injection on the same schedule. All animal experiments complied with the Guidelines for Ethical Review of Laboratory Animal Welfare (GB/T 35892-2018) and were approved by the Ethics Committee of Guangdong Provincial Hospital of Traditional Chinese Medicine (Ethics Approval No. 2022025).

### 2.10. Sample Collection

Mice were fasted for 12 h prior to the final treatment. Blood was collected from the eyeball, allowed to stand for 30 min, and centrifuged at 3500 rpm for 20 min to separate the serum. After cervical dislocation, the liver, spleen, and kidneys were weighed. The left hind limb was collected, fixed in 10% paraformaldehyde, subjected to micro-CT analysis, and then decalcified in EDTA solution for 3 weeks. The samples were embedded, sectioned, and subjected to histopathological examination. The right hind limb was evenly divided into two portions: one portion was placed in a cryovial with 1 mL RNA preservation solution, and the other was placed in a separate cryovial. Both vials were stored in liquid nitrogen for 2 h before being transferred to a −80°C freezer for subsequent gene and protein analyses.

### 2.11. General Condition and Joint Swelling Assessment

The overall condition, fur condition, and activity level of the mice were observed. The weight and joint swelling of each group of mice were recorded weekly. Before the model induction, a mark was made 0.5 cm below the ankle joint, and the width and thickness of the mark were measured using a vernier caliper. After the model induction, the joint swelling was measured at the same location weekly, and the AI score was detected. The detailed rules of the AI score: Joint swelling was scored by two independent blinded investigators using a 0–4 scale: 0 = *no redness or swelling, normal appearance of the joints, and free movement*; 1 = *mild swelling of the joints, possibly limited to a single toe or the little toe joint, with basically unrestricted joint movement*; 2 = *moderate swelling of the joints, possibly involving multiple toes or metacarpophalangeal joints, with slightly restricted joint movement*; 3 = *severe swelling of the joints, involving the entire sole or ankle joint, with significantly restricted joint movement*; 4 = *severe swelling of the joints, even involving the entire foot up to the ankle joint above, with complete loss of joint function, and possible stiffness or deformity*.

### 2.12. Pathological Evaluation

#### 2.12.1. Micro-CT

After the modeling process was completed, mice were fasted for 12 h. The left hind limb was collected, soft tissues were removed, and the limb was fixed in 10% paraformaldehyde. A desktop micro-CT system (Bruker) was used to scan the left hind limb. Cross-sectional analysis was performed using CT Vox software, and three-dimensional reconstruction was conducted using NRecon software (Version 1.6.10.4; Bruker).

#### 2.12.2. H&E Staining and Safranin O-Fast Green Staining

After micro-CT analysis, the left hind limb was transferred to a 20-fold volume of EDTA decalcification solution for 3 weeks. Following decalcification, the samples were embedded, sectioned, and subjected to H&E staining and Safranin O-Fast Green staining to evaluate bone tissue pathological damage.

### 2.13. Western Blot

Joint tissues from mice were homogenized in RIPA lysis buffer to extract proteins, and total protein concentration was determined using a BCA protein assay kit. Protein samples were separated by 8%–12% SDS-PAGE and transferred to a PVDF membrane (Bio-Rad) using wet electrotransfer. The membrane underwent a blocking process in Tris-Tween buffered saline (TBST) with 5% non-fat dry milk for 2 h at room temperature, followed by overnight incubation at 4°C with the following primary antibodies: RUNX2 (CST 12556, 1:1000), OPN (CST 88742, 1:1000), SOX (CST 82630, 1:1000), and *β*-actin (Proteintech 20536-1-AP, 1:4000). Following three washes with TBST, the membrane was incubated with secondary antibodies for a duration of 2 h at ambient temperature. The visualization of protein bands was achieved through an enhanced chemiluminescence detection system, with ImageJ software used to quantify the grayscale values.

### 2.14. RT-qPCR

Total RNA was extracted from joint tissue samples using an RNA extraction kit. After assessing the purity and concentration of the RNA, it was reverse transcribed into cDNA and subjected to real-time PCR reactions to detect the mRNA expression of *β-actin*, *RIOK1*, *FUCA2*, *COL9A2*, *USP16*, and *TTC16*. The PCR process began with an initial denaturation phase at 95°C lasting 15 min, followed by 40 cycles consisting of denaturation at 95°C for 10 s, annealing at 58°C for 30 s, and extension at 72°C for 30 s. *β*-Actin served as the internal control, and the relative expression levels of mRNA were determined using the 2 − ΔΔCt method for relative quantification. The sequences of the primers utilized are provided in Table 1.

### 2.15. Statistical Analysis

Statistical analyses and data visualization were conducted using R Version 4.2. Between-group comparisons of gene expression profiles and immune cell infiltration patterns across clinical subgroups were evaluated through nonparametric Wilcoxon rank-sum tests (two-tailed). Relationship assessments between variables were performed using Spearman's rank correlation coefficient.

## 3. Results

### 3.1. Identification Results of AS DEGs

**Figure 2:**
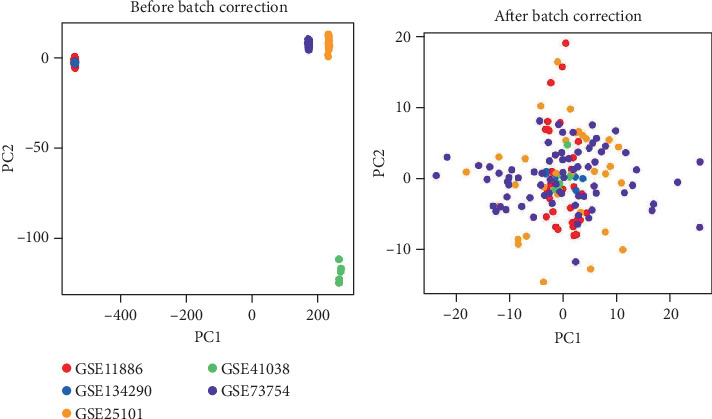
Scatter plot of principal component analysis before and after batch effect removal in the GEO dataset.

We retrieved five datasets (*GSE11886*, *GSE134290*, *GSE25101*, *GSE41038*, and *GSE73754*) from the GEO database. By performing batch effect correction, we reduced the batch effects between different datasets (Figure 2). We then conducted differential expression analysis between the AS and control groups using the integrated dataset, identifying 1607 DEGs with significant differences. The gene expression and differential analysis results are provided in Supporting Information 1: File S1.

### 3.2. Results of MR Analysis

Based on the differential analysis, 1366 *cis*-eQTLs were matched for the 1607 DEGs on the eQTLGen Consortium, with instrumental variables provided in Supporting Information 2: File S2. After the Bonferroni correction, the significance threshold for MR analysis was *p* = 1.83 × 10−^−5^ (0.05/2732). The MR analysis results of the DEGs with the AS training and validation sets are provided in Supporting Information 3: File S3 and Supporting Information 4: File S4. After excluding results with significant heterogeneity and horizontal pleiotropy, 248 DEGs were obtained from the training set, and 229 DEGs were obtained from the validation set, with 40 DEGs showing causal relationships with AS after taking the intersection (Supporting Information 5: File S5).

After the Bonferroni correction, the significance threshold for SMR analysis was *p* = 1.25 × 10^−3^ (0.05/40). The SMR analysis results are presented in Supporting Information 6: File S6, showing that all 40 DEGs had causal associations with AS, but seven DEGs did not pass the HEIDI test (*p* < 0.01), indicating linkage disequilibrium, and were therefore excluded. Ultimately, 33 DEGs with causal relationships with AS were identified as Positive DEGs.

**Figure 3:**
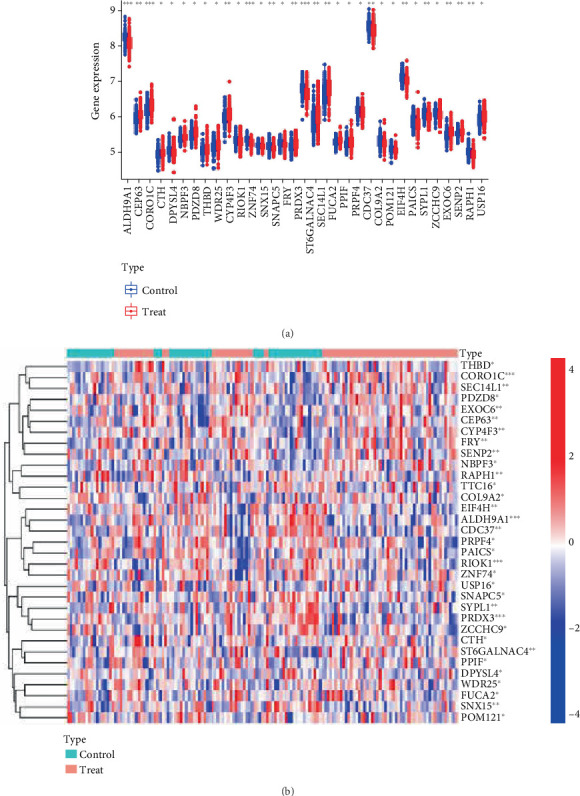
(a) Box plot of AS-related differential gene expression analysis. (b) Heatmap of AS-related differential gene expression analysis.

The differential expression analysis results of the 33 Positive DEGs are shown in Figures 3a,b. The results indicate that *ALDH9A1*, *DPYSL4*, *TTC16*, *RIOK1*, *ZNF74*, *SNX15*, *SNAPC5*, *PRDX3*, *PRPF4*, *CD37*, *COL9A2*, *POM121*, *EIF4H*, *PAICS*, *SYPL1*, *ZCCHC9*, and *RAPH1* were decreased in the AS group, while *CEP63*, *CORO1C*, *CTH*, *NBPF3*, *PDZD8, THBD*, *WDR25*, *CYP4F3*, *FRY*, *ST6GALNAC4*, *SEC14L1*, *FUCA2*, *PPIF*, *EXOC6*, *SENP2*, and *USP16* were overexpressed in the AS group.

### 3.3. Correlation Analysis Between Positive DEGs and Immune Cell Expression

**Figure 4:**
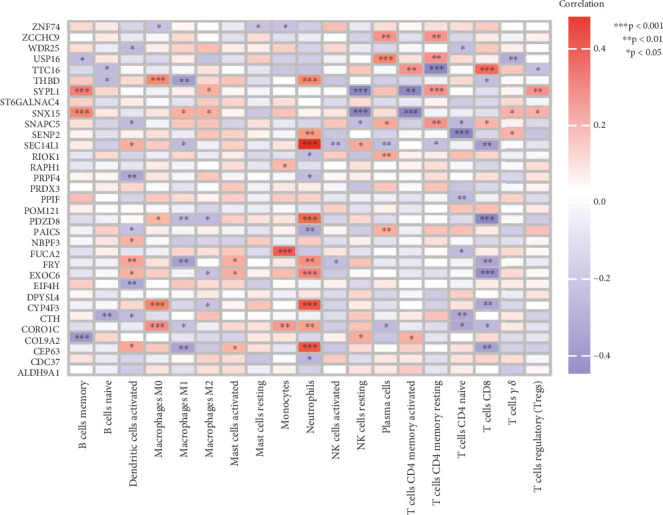
Heat map of correlation analysis.

We identified DEGs with causal relationships to AS through MR analysis, followed by validation with SMR, and then analyzed the correlation between the expression of Positive DEGs and immune cell abundance to reveal the potential regulatory relationships between these genes and immune cells. As shown in Figure 4, 28 out of the 33 positive DEGs showed significant regulatory relationships with 19 immune cell types. Among these significant gene-immune cell pairs, 55 showed negative regulatory relationships and 49 showed positive regulatory relationships.

### 3.4. Identification of AS Signature Genes and Machine Learning Model Construction Results

**Figure 5:**
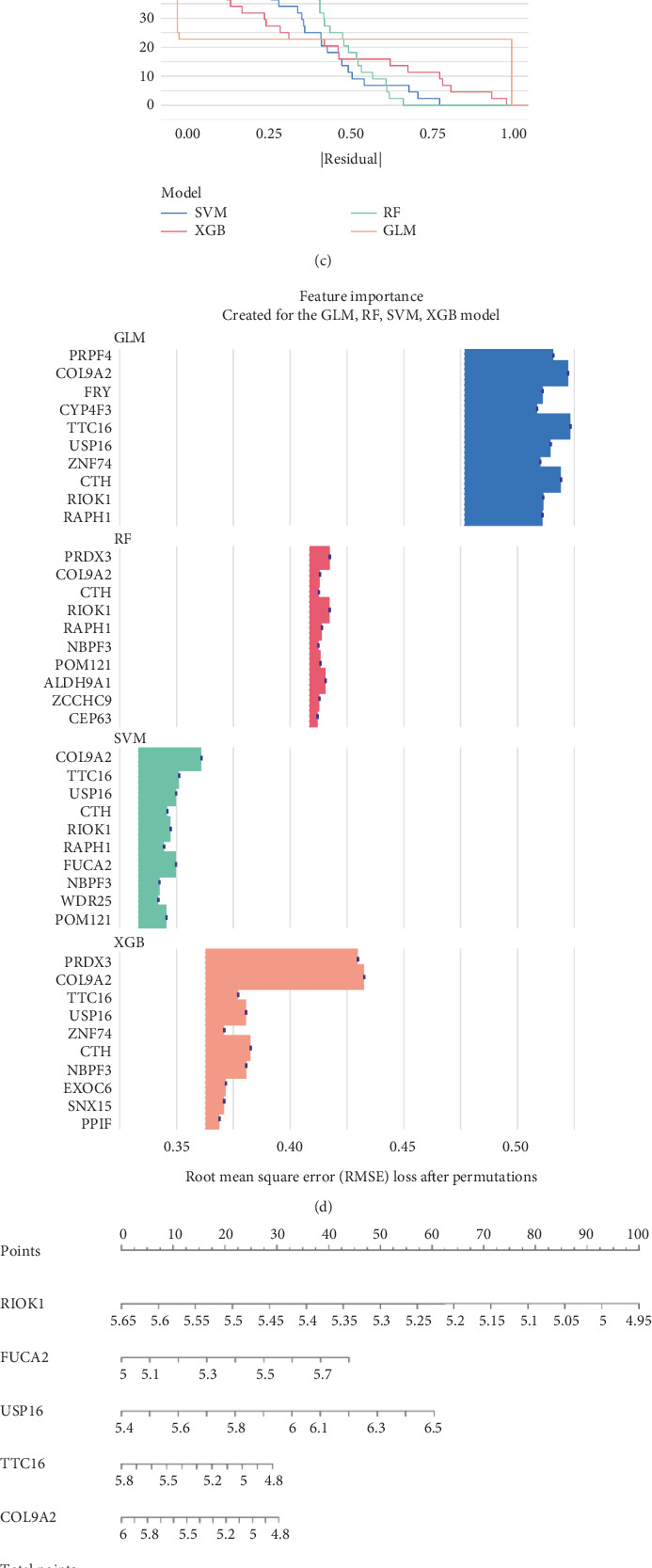
(a) ROC of the four machine learning models. (b) Box plots of residuals of the four machine learning models. (c) Reverse cumulative distribution plot of residuals for the four machine learning models. (d) Bar plot of feature importance of the four machine learning models. (e) Nomogram of the feature genes. (f) Decision curve of feature genes nomogram. (g) Calibration curve of feature genes nomogram.

As shown in Figures 5a, 5b, and 5c, among the four machine learning models we constructed, the SVM method demonstrated the highest area under the ROC curve and the lowest residuals. Therefore, we selected this model for further analysis and determined the importance scores of the signature genes (Figure 5d). The Top 5 genes with the highest importance scores used for constructing the nomogram were *RIOK1*, *FUCA2*, *COL9A2*, *USP16*, and *TTC16*. Subsequently, we obtained scoring scales for these five signature genes (Figure 5e). By summarizing the expression scores of the signature genes, we assessed the risk probability of AS based on the causally related signature genes. The accuracy of the model is reflected in the decision curve by the distance between the red and gray lines (Figure 5f,g).

### 3.5. PheWAS Results of AS Signature Genes

The results of the PheWAS are provided in Supporting Information 7: Files S7–S11. After the Bonferroni correction, the significance threshold for the PheWAS analysis was *p* = 1.27 × 10^−5^ (0.05/3948). Based on the PheWAS analysis results of the five AS signature genes, we filtered out all positive disease phenotypes and excluded results with significant heterogeneity and horizontal pleiotropy. Among them, *TTC16* showed harmful effects on 27 disease phenotypes across 9 disease categories and beneficial effects on 36 disease phenotypes across 9 disease categories; *USP16* had harmful effects on 40 disease phenotypes across 9 disease categories and beneficial effects on 66 disease phenotypes across 10 disease categories; *RIOK1* showed harmful effects on 33 disease phenotypes across 8 disease categories and beneficial effects on 31 disease phenotypes across 8 disease categories; *FUCA2* had harmful effects on 69 disease phenotypes across 10 disease categories and beneficial effects on 51 disease phenotypes across 11 disease categories; *COL9A2* showed harmful effects on 22 disease phenotypes across 7 disease categories and beneficial effects on 24 disease phenotypes across 7 disease categories.

### 3.6. Molecular Docking Results of AS Signature Genes

**Figure 6:**
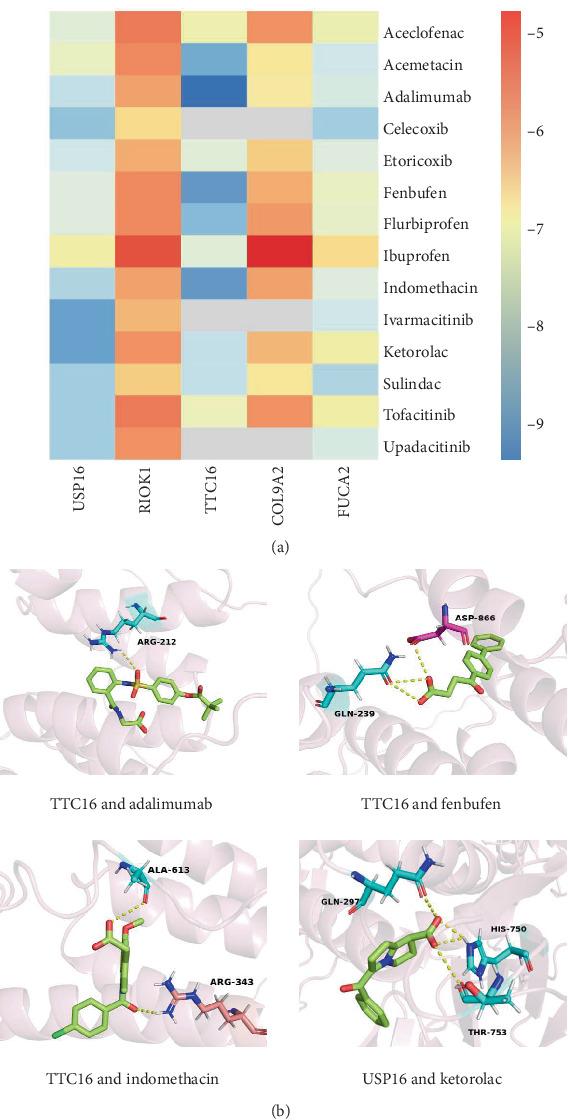
(a) Heatmap of binding energies from molecular docking between AS signature genes and 14 AS therapeutic drugs. (b) Molecular docking visualization.

We collected 14 known therapeutic drugs for AS from DrugBank, including Aceclofenac, Acemetacin, Adalimumab, Celecoxib, Etoricoxib, Fenbufen, Flurbiprofen, Ibuprofen, Indomethacin, Ivarmacitinib, Ketorolac, Sulindac, Tofacitinib, and Upadacitinib. To verify whether there are potential interactions between AS therapeutic drugs and AS causal DEGs, we performed molecular docking. The molecular docking results showed that the binding energies of the majority (88.6%) of the docking combinations were below −5.0 kcal/mmol, indicating that stable structures could form, suggesting potential therapeutic effects (Figure 6a).

The top four docking results are presented in Figure 6b. Among them, Adalimumab primarily interacts with USP16 through hydrogen bonds with residues ASP866, ARG21, LEU243, GLN239, and PHE209, resulting in a strong binding energy of −9.0 kcal/mmol. Fenbufen interacts with USP16 via hydrogen bonds involving residues TYR870, TYR174, MET177, LEU181, and ASP866, with a binding energy of −8.5 kcal/mmol. Indomethacin docks with USP16 via a hydrophobic pocket, resulting in a binding energy of −8.5 kcal/mmol, involving residues such as LEU181, PHE209, LEU867, and LEU858. Additionally, Ketorolac can bind to TTC16 via hydrogen bonds involving residues TYR759, HIS750, PHE700, LEU581, and LEU585, with a binding energy of −8.4 kcal/mmol.

### 3.7. General Observations in AS Model Mice

**Figure 7:**
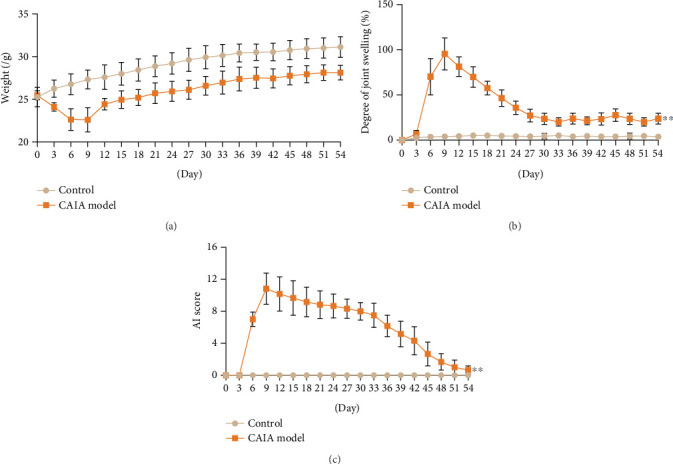
(a) Body weight in each group. (b) Degree of joint swelling. (c) Total AI score in each group. ⁣^∗∗^*p* < 0.01 compared with control group.

As shown in Figure 7, 3 days after modeling, the AS mice exhibited reduced body weight, paw swelling, lethargy, and decreased food and water intake compared with the control group. Subsequently, joint swelling progressively worsened, peaking on Day 9, accompanied by reduced mobility. Thereafter, body weight gradually increased, while joint swelling significantly decreased (*p* < 0.01). Compared with the control group, the AI score in the model group was significantly elevated. However, as inflammation naturally subsided, joint swelling gradually alleviated (*p* < 0.01).

### 3.8. Pathological Evaluation of AS Model Mice

**Figure 8:**
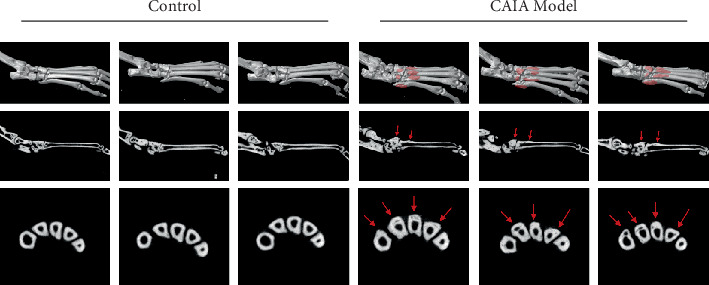
Micro-CT in each group. The pathological new bone formation area is marked in red.

**Figure 9:**
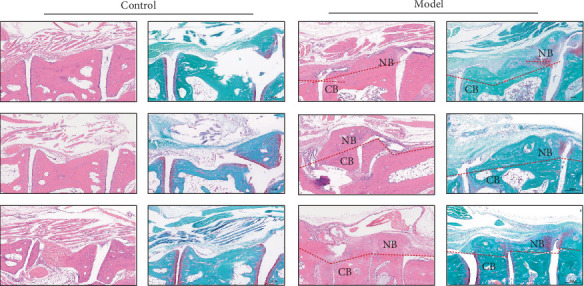
H&E staining and Safranin O-Fast Green staining (100x). NB, new bone; CB, cortical bone.

As shown in Figure 8, micro-CT analysis revealed ectopic new bone formation at the tendon–bone junctions of the tarsal and phalangeal bones in the model group, along with bone erosion along the inner bone marrow cavities, compared with the control group. As shown in Figure 9, H&E staining further demonstrated thickening of the tarsal and metatarsal bones, as well as their articular surfaces. Safranin O-fast green staining indicated that the original ligament tissue attached to the bone and joint surfaces had differentiated into red-stained osseous and cartilaginous tissues, suggesting pathological ossification.

### 3.9. Bone Mineralization Factors in AS Model Mice

**Figure 10:**
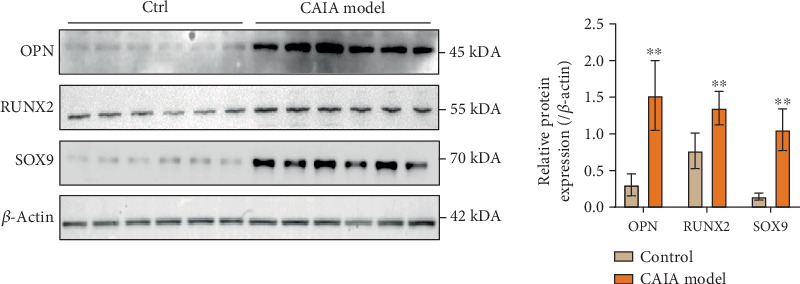
Changes of the protein expression of OPN, RUNX2, SOX9, and *β*-actin in joint tissue, ⁣^∗∗^*p* < 0.01 compared with control group.

As shown in Figure 10, Western blot analysis revealed that the protein expression levels of RUNX2, OPN, and SOX9 in the left hind paw tissues of the model group were significantly higher than those in the control group (*p* < 0.01), indicating an active pathological ossification process.

### 3.10. Functional Validation Results of AS Signature Genes

**Figure 11:**
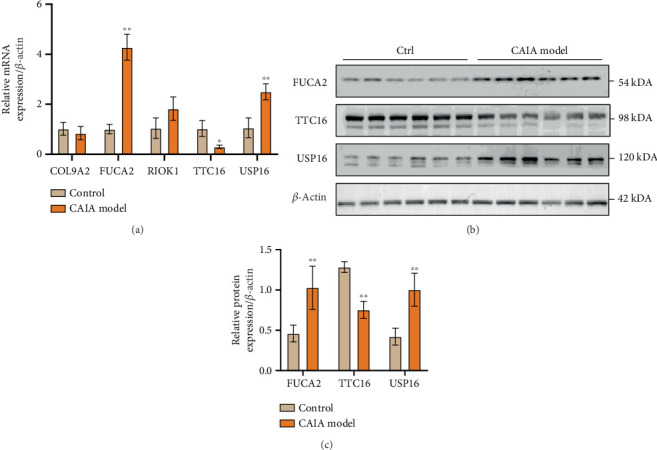
(a) The mRNA expression levels of key genes in joint tissue. (b) Changes in the protein expression of FUCA2, TTC16, USP16, and *β*-actin in joint tissue. (c) The quantified grayscale value. ⁣^∗∗^*p* < 0.01 compared with the control group.

As shown in Figure 11, compared with the control group, the mRNA levels of *FUCA2* and *USP16* in the model group were significantly increased (*p* < 0.01), and the mRNA level of *TTC16* was decreased (*p* < 0.05) in the model group. However, there was no significant difference in the expression of *COL9A2* and *RIOK1*. We further conducted WB analysis to detect the protein expression levels of FUCA2, TTC16, and USP16. It was found that compared with the normal group, the protein expression levels of FUCA2 and USP16 in the CAIA model group were increased (*p* < 0.01), while the protein expression level of TTC16 was decreased (*p* < 0.01).

## 4. Discussion

AS is a chronic inflammatory disorder primarily impacting the axial joints, with a global prevalence of 0.1% to 1.4% [9]. It is marked by inflammation of sacroiliac joints and spinal entheses, causing inflammatory back pain in early stages. Over time, AS can lead to joint ankylosis, spinal deformities, and interstitial lung disease, reducing patients' quality of life [10]. The human leukocyte antigen B27 (HLA-B27) allele is strongly linked to AS, but over 90% of HLA-B27-positive individuals do not develop the disease, indicating that environmental factors, epigenetics, and gene-environment interactions play key roles in AS development [11]. Current treatments, such as NSAIDs and tumor necrosis factor-*α* (TNF-*α*) inhibitors, are ineffective for 30%–40% of patients, and they do not prevent structural damage like bone erosion or new bone formation [12]. Thus, understanding the molecular mechanisms of AS and finding targeted therapies are critical. This study combines transcriptomics, MR, and machine learning to identify potential therapeutic targets for AS and assess their suitability for drug development.

By analyzing five GEO datasets, we identified 1607 DEGs. MR and summary data–based MR analyses confirmed 33 DEGs with causal links to AS. MR used SNPs as instrumental variables to simulate randomized controlled trials, reducing bias and providing stronger evidence of gene-disease connections than traditional studies. For example, ALDH9A1 and TTC16 were downregulated in AS patients, while FUCA2 and USP16 were upregulated, suggesting their roles in AS through key biological processes. These findings were validated in FinnGen and UK Biobank datasets, addressing concerns about heterogeneity and pleiotropy.

Correlation analysis showed that 28 of the 33 DEGs regulated 19 immune cell types, forming 55 negative and 49 positive regulatory pairs. This suggests that AS-related immune dysregulation stems from gene-mediated changes in immune cell function or differentiation. Previous studies highlighted Th1/Th17 cell hyperactivation in AS, but our results map a detailed gene–immune cell network, showing how gene expression shapes the immune microenvironment during AS progression. These findings indicate that immune cell-mediated gene expression changes are a key mechanism in AS, offering new targets for immune-based therapies.

Using four machine learning models, including the RF model, SVM model, GLM model, and XGBoost model, we identified RIOK1, FUCA2, COL9A2, USP16, and TTC16 as key genes. The SVM model performed best, with the highest AUC and lowest residuals. A nomogram based on these five genes accurately predicted AS risk, providing a tool for early diagnosis. Notably, FUCA2 and USP16 were upregulated, and TTC16 was downregulated in AS patients, consistent with patterns in CAIA mouse models, supporting their use as biomarkers. These genes enable AS risk prediction models, aiding early diagnosis and prognosis.

PheWAS analysis showed that the five key genes were linked to various disease phenotypes. For example, FUCA2 had harmful effects on 69 phenotypes, while TTC16 had beneficial effects on 36 phenotypes, highlighting the need to assess potential side effects in drug development. PheWAS analysis unveiled significant associations between FUCA2 and multiple immunopathological phenotypes, with particularly notable positive correlations observed in rheumatoid arthritis and inflammatory bowel disease. These conditions share immunopathogenic mechanisms with AS, including dysregulated IL-23/Th17 axis activation and osteo-immune dysbalance. These findings collectively identify FUCA2 and TTC16 as a novel molecular entry point for targeting pathological bone remodeling in immune disease. Molecular docking simulations showed stable binding between 14 AS drugs and these genes, identifying candidates for further drug development.

To verify the function of the characteristic genes, we established a CAIA mouse model. Animal models play a pivotal role in unraveling the pathomechanisms of AS and evaluating therapeutic interventions. The CAIA model was strategically selected for its unique capacity to recapitulate spontaneous entheseal heterotopic ossification—a cardinal pathological feature of AS. This model is induced through systemic administration of antitype II collagen monoclonal antibodies followed by LPS challenge, thereby phenocopying the dual inflammatory-osteogenic cascade observed in AS pathogenesis [13]. Histopathological analysis of CAIA mice revealed AS-like features including cartilaginous metaplasia, trabecular bone formation, and periosteal hyperostosis in affected joints, demonstrating striking phenotypic convergence with human syndesmophyte development [14]. In contrast to TNF-*α*-driven models that predominantly exhibit inflammatory arthritis with synovial hyperplasia and cartilage erosion but minimal osteoproliferation, the CAIA paradigm uniquely manifests concurrent synovitis and intra-articular ossification. This immuno-osseous pathology closely mirrors the entheseal bone formation observed in AS patients, rendering the CAIA model particularly advantageous for investigating immune-mediated osteogenesis in this study. We observed joint swelling, ectopic bone formation, and increased expression of bone mineralization factors RUNX2 and OPN, confirming the model's validity. RT-qPCR results showed FUCA2 and USP16 upregulation and TTC16 downregulation, matching bioinformatics findings and validating these genes' roles in inflammation, cartilage matrix balance, and bone remodeling.

FUCA2 encodes plasma *α*-L-fucosidase, a glycosyl hydrolase that removes *α*-1,6-linked fucose from glycoproteins and glycolipids [15]. This process regulates immune responses, especially leukocyte movement and adhesion. High *α*-L-fucosidase activity in chronic inflammatory diseases [16] suggests FUCA2 contributes to AS. Specifically, FUCA2 overexpression may increase immune cell recruitment to axial joints by enhancing defucosylation, promoting chronic inflammation [17]. Additionally, altered glycosylation may disrupt extracellular matrix (ECM) interactions and bone signaling [18], worsening AS progression.

USP16, a deubiquitinase, regulates cell cycle progression and gene expression. It plays a pivotal role in autoimmune inflammation, particularly by modulating the NF-*κ*B signaling pathway. A study demonstrated that USP16 enhances NF-*κ*B activation by selectively removing K238-linked ubiquitination from IKK*β*, thereby promoting p105 phosphorylation and exacerbating inflammatory responses in inflammatory bowel disease (IBD) [19]. The expression of USP16 is significantly elevated in colonic macrophages of patients suffering from IBD and is positively associated with disease activity. Importantly, mice with a knockout of USP16 display a reduction in colitis severity, characterized by decreased levels of pro-inflammatory cytokines such as TNF, IL-12, and IL-23. In the context of gouty arthritis, USP16 contributes to the inflammatory process through drp1-dependent mitochondrial fission and the activation of the NLRP3 inflammasome [20]. The sustained activation of the canonical NF-*κ*B pathway, in which USP16 is instrumental, is strongly associated with osteoproliferation in AS [21]. Furthermore, USP16 influences T-cell activation and proliferation by regulating calcineurin A activity, reinforcing its role in autoimmune diseases [22]. Thus, the elevated USP16 expression in AS positions it as a key pro-inflammatory mediator and a promising therapeutic target for further investigation.

TTC16 encodes tetratricopeptide repeat (TPR) Domain 16, a protein-coding gene. TPR domains support protein–protein interactions, which are essential for signal transduction, assembling protein complexes, or regulatory mechanisms [23]. Given their role in signal transduction, TTC16 likely modulates the inflammatory milieu of AS and osteocyte signaling by interacting with immune-related proteins, cytokine receptors, or transcription factors. Additionally, the TTC protein family is integral to the formation and function of primary cilia [24] which are present on osteoblasts, osteocytes, and chondrocytes. These cilia detect extracellular chemical and mechanical signals, orchestrating cellular responses. Primary cilia are essential for skeletal and cartilage development, modulating mechanotransduction and managing bone remodeling along with cartilage matrix formation [25]. In AS, pathological ossification occurs at sites of mechanical stress. Disruption of cilium-associated protein complexes may alter the capacity of bone and cartilage cells to sense and respond to mechanical or inflammatory signals, thereby promoting aberrant bone formation and chronic inflammation [26].

Additionally, COL9A2 and RIOK1 were linked to bone mineralization pathways, correlating with ectopic bone formation in AS. Western blot results confirmed higher RUNX2 and OPN expression in AS models, supporting their role in bone abnormalities.

This study integrates transcriptomic profiling, MR, machine learning, and animal model validation to identify therapeutic targets for AS. Transcriptomic analysis identified DEGs, MR established causal associations, machine learning optimized feature gene selection, and animal experiments validated targets. This multi-omics approach mitigates the limitations of single-method analyses, enhancing the robustness of target identification. Specifically, FUCA2, USP16, and TTC16 emerged as candidate biomarkers for AS.

Limitations of this study include the use of public database-derived data, which may introduce population heterogeneity and batch effects. Future investigations should increase sample sizes and validate findings in independent cohorts. Additionally, the functional mechanisms of AS-associated feature genes remain underexplored. Targeted gene knockout or overexpression studies are needed to elucidate their roles. Furthermore, while PheWAS identified pleiotropic effects of AS feature genes, their phenotypic associations require validation in prospective clinical cohorts.

## 5. Conclusion

This study integrates multi-omics data with machine learning to identify critical pathogenic genes and therapeutic targets for AS. Specifically, FUCA2, USP16, and TTC16 contribute to AS pathogenesis, immune cell regulation, and drug target interactions, offering candidate biomarkers and strategies for precise AS diagnosis and therapy.

## Figures and Tables

**Table 1:** Primer sequences used for rat target genes.

**Genes**	**Primer sequence (5**⁣′**-3**⁣′**)**
*β-actin*-mouse-F	AGGCCAACCGTGAAAAGATG
*β-actin*-mouse-R	ATGCCAGTGGTACGACCAGA
*RIOK1*-mouse-F	GCAGAGGTGAAGGGCGAAGATG
*RIOK1*-mouse-R	AGTCGAGTCGTCCAAGTACCAGTC
*FUCA2*-mouse-F	GCTTGAGGTGGCTGTGAGGAAC
*FUCA2*-mouse-R	GGGAAACCGCTGCTTCTGGAAC
*COL9A2*-mouse-F	ACGGCAAGGATGGAGACAGAGG
*COL9A2*-mouse-R	TGAGGCGAGCAGAGGTATAGGC
*USP16*-mouse-F	TCAGGGCTGTGGCAGAGATTCC
*USP16*-mouse-R	CACTTGTAACACCAGACGCTCCAG
*TTC16*-mouse-F	CAGCCACCGCCGTGACATTC
*TTC16*-mouse-R	GCTTAGTGCCTCCCTGTTGTTCC

## Data Availability

The data that support the findings of this study are available from the corresponding author upon reasonable request.

## Supporting Information

Supporting Information 1 File S1: The gene expression and differential expression analysis results identifying 1607 DEGs between the AS and control groups.

Supporting Information 2 File S2: The instrumental variables for 1366 cis-eQTLs matched to the DEGs based on the eQTLGen Consortium.

Supporting Information 3 File S3: The MR analysis results for DEGs using the AS training set.

Supporting Information 4 File S4: The MR analysis results for DEGs using the AS validation set.

Supporting Information 5 File S5: The 40 DEGs with causal relationships to AS, identified after excluding results with significant heterogeneity and horizontal pleiotropy from the training and validation sets.

Supporting Information 6 File S6: The SMR analysis results for the 40 DEGs.

Supporting Information 7 Files S7–S11: The PheWAS results for the five AS signature genes.
